# Pressure as a Preventive of Inflammation

**Published:** 1861-01

**Authors:** 


### PRESSURE AS A PREVENTIVE OF INFLAMMATION.

Dr. Cooper, of the San Francisco *Medical Press*, makes the following judicious remarks on this subject:

"We have long been in the habit of applying a roller, as tightly as the patient could bear, over the extremities, to prevent inflammation, in cases of operations for pseudarthrosis, exsections of bones, or accidental injuries.

"Not only can compression be relied upon to keep down inflammation before it is established, but painful parts are often relieved by it.

"Such is our confidence in the efficacy of pressure in preventing inflammation after injuries, that there are many operations we are constantly performing successfully that would not be attempted at all, were it not that we can anticipate and prevent inflammation by pressure, applied by means of rollers. Its reliability and efficiency render it invaluable.

"How can there be increased redness or swelling without an increase of blood in the parts? And if there is no swelling or flow of blood to the part, the heat and pain will be prevented. So that it is easy to perceive that, by preventing the blood from flowing into the part, we prevent the inflammation.

"Even after inflammation is established, pressure will often be found not only unirritating, when applied to the arterial trunk supplying it, but directly to the part itself."

